# Interaction between opium use and cigarette smoking on bladder cancer: An inverse probability weighting approach based on a multicenter case-control study in Iran

**DOI:** 10.1016/j.gloepi.2024.100182

**Published:** 2024-12-30

**Authors:** Rahim Akrami, Maryam Hadji, Hamideh Rashidian, Maryam Nazemipour, Ahmad Naghibzadeh-Tahami, Alireza Ansari-Moghaddam, Kazem Zendehdel, Mohammad Ali Mansournia

**Affiliations:** a Department of Epidemiology and Biostatistics, School of Public Health, Tehran University of Medical Sciences, Tehran, Iran; b A.I. Virtanen Institute for Molecular Sciences, University of Eastern Finland, Kuopio 70150, Finland; c Cancer Research Center, Cancer Institute, Tehran University of Medical Sciences, Tehran, Iran; d Social Determinants of Health Research Center, Institute for Futures Studies in Health, Kerman University of Medical Sciences, Kerman, Iran; e Department of Biostatistics and Epidemiology, Kerman University of Medical Sciences, Kerman, Iran; f Health Promotion Research Center, Zahedan University of Medical Sciences, Zahedan, Iran; g Cancer Biology Research Center, Cancer Institute of Iran, Tehran University of Medical Sciences, Tehran, Iran

**Keywords:** Additive interaction, Bladder cancer, Case control, Cigarette smoking, Mechanistic interaction, Opium using

## Abstract

**Introduction:**

Opium and cigarette smoking have been identified as significant cancer risk factors. Recently, the International Agency for Research on Cancer (IARC) classified opium as a Group 1 carcinogen in 2020.

**Method:**

Using data from a multicenter case-control study in Iran called IROPICAN, involving 717 cases of bladder cancer and 3477 controls, we assessed the interactions on the causal additive scale between opium use and cigarette smoking and their attributing effects to evaluate public health relevance and test for different mechanistic interaction forms to provide new insights for developing of bladder cancer. A minimally sufficient set of confounders was identified using a causal directed acyclic graph, and the data were analysed employing multiple logistic regression and the inverse probability-of-treatment weighting estimator of the marginal structural linear odds model.

**Results:**

Our findings indicated a significant increase in the risk of bladder cancer associated with concurrent opium use and cigarette smoking (adjusted OR = 6.34, 95 % CI 5.02–7.99; *p* < 0.001), demonstrating a super-additive interaction between these exposures (Weighted RERI_OR_ = 2.02, 95 % CI 0.47–3.58; *p* = 0.005). The presence of a super-additive interaction suggests that interventions targeting opium users who smoke cigarettes would yield greater benefits compared to non-opium users. Furthermore, there was a mechanistic interaction between two exposures (*P*-value = 0.005) if we assumed two of the exposures have positive monotonic effects, i.e., there must be a sufficient-component cause for developing bladder cancer, which has both opium use and cigarette smoking as components.

**Conclusion:**

There is a causal additive interaction between opium use and cigarette smoking. We observed a super-additive interaction, suggesting the need to focus interventions on specific subgroups. Furthermore, the presence of mechanistic interactions offers profound insights into the mechanisms of cancer induction.

## Highlights

• The inverse probability-of-treatment weighting method used to estimate interaction.
• Mechanistic interactions were assessed by two approaches.
• The presence of super-additive causal interaction between opium and smoking.
• Targeting opium-smoking users could maximize intervention benefits.

## Introduction

Bladder cancer is the 6th most common cancer in the world and 3rd in Easten Mediterranean Regional (EMR) with a 10.4 per 100,000 age-standardized incidence rate among males. This cancer is four times more common in men than in women [1]. In Iran, the incidence of this cancer is increasing and it accounts for approximately 7 % of all cancers [2]. Bladder cancer is associated with multiple risk factors, with tobacco use being the predominant one, followed by Schistosoma haematobium infection, and occupational exposures, as well as arsenic in drinking water, while lifestyle and dietary factors also contribute to its etiology [3,4]. Recent studies indicate that the risk of bladder cancer for those who smoke is greater than for most other types of cancer, except the head, neck, and respiratory system [5].

Opium is the dried extract of opium poppy, and Iran accounts for 42 % of its consumption worldwide [6]. Opium is being discussed and investigated as one of the important cancer risk factors, and the International Agency for Research on Cancer (IARC) categorized it as the group I carcinogen in 2020 [[7], [8], [9], [10], [11]].

In recent years, there has been a growing focus on studying how environmental exposures interact with each other [12]. Despite the significance of this area of study, it has received relatively little attention in public health and health policy due to the complexities involved in interaction issues and their calculations [13,14]. Also, relying only on interaction measures in a multiplicative scale is inadequate for the assessment of the public health implications of exposure interactions on cancer development [13,15]. On the other hand, there are some concerns regarding the accuracy of case-control studies in estimating a causal additive interaction by delta-method-based approaches to RERI (Relative Excess Risk due to Interaction) [[16], [17], [18]] and test sufficient-cause interactions through outcome regression models [19]. As a result, the available evidence is insufficient to fully understand the causal interaction between opium use and cigarette smoking.

We aimed to assess all measures of interactions in the causal additive scale between opium use and cigarette smoking and their attributing effects to evaluate public health relevance (how best to allocate interventions) and test mechanistic interactions to provide new insights into the mechanisms for the development of bladder cancer by multicenter case-control study and an inverse probability weighting approach.

## Methods

### Study design and setting

We used data from the IROPICAAN study [20], a multicenter case-control study conducted across ten provinces in Iran and studied the risk of opium use and the risk of four cancer sites, including lung, bladder, colorectal and head and neck cancers. Cases were 717 histologically confirmed bladder cancer patients (ICD-O: C67) who were admitted between May 2017 and July 2020 in referral hospitals. All cases were newly diagnosed within one year before the interview. The interviews were conducted using a validated questionnaire from the IROPICAN study [21].

The controls consisted of 3477 healthy visitors who were selected from among the relatives or friends of patients who did not have cancer from wards not associated with oncology. Controls were frequency-matched based on place of residence (by province and capital city/non-capital city), age (five-year interval) and sex. They were cancer-free subjects and accompanied hospitalized patients in the non-oncology unit.

### Confounder identification

**Fig. 1:**
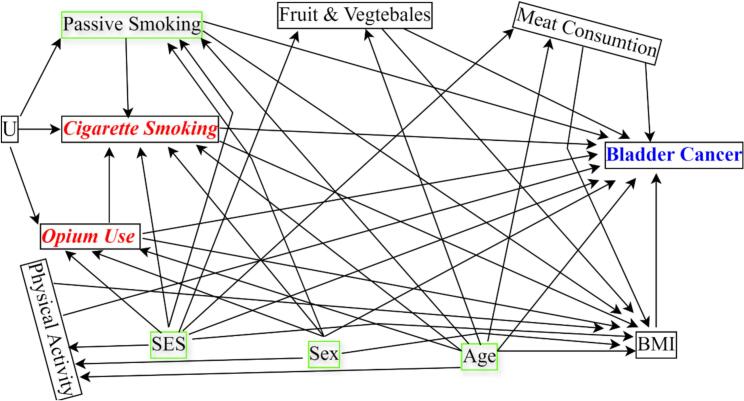
A causal directed acyclic graph in which opium use and cigarette smoking are exposures and bladder cancer is the outcome; U1 is an unmeasured confounder; SES is socio-economic status; Green boxes represent a minimally sufficient set of confounders. (For interpretation of the references to colour in this figure legend, the reader is referred to the web version of this article.)

The confounders for both effects of opium use and cigarette smoking on bladder cancer were adjusted to evaluate causal additive interaction [22,23]. The browser-based tool (DAGitty) was used to draw a causal directed acyclic graph (cDAG) for the study setting. Subsequently, a minimally sufficient set of confounders was identified: age, sex, passive smoking, and socio-economic status of the subjects (Fig. 1) [[24], [25], [26], [27], [28], [29], [30], [31], [32], [64], [65], [66]].

Various factors associated with socioeconomic status (SES) were consolidated through principal component analysis by combining data on years of education, and ownership of vacuum cleaner, dress washing machine, dishwashing machine, freezer, microwave, split air conditioner, laptop, internet access, mobile phone, personal car, owned house, or a shop. The weighted sums of these variables, with weights corresponding to the principal component loading, were divided into tertiles and utilized as the SES variable in the regression models.

We estimated perceived physical activity workload (PPWL) by analysing the job histories of participants alongside a Finnish job exposure matrix (FINJEM) [22,23]. FINJEM includes two variables for estimating PPWL for each occupation and time period: the proportion of exposed individuals (P) and the average level of exposure among those exposed (L) [24]. Due to overlapping work periods in the data we collected, we were unable to calculate the cumulative PPWL for an individual's entire work life. As a result, we focused on PL for the longest work period, which we classified into three categories: sedentary, moderate, or heavy (the highest tertile of non-sedentary observations).

Sensitivity analyses were performed by adding the matched province variable (with ten levels) to the set of confounders and assessment based on the change-in-estimate criterion with a threshold of 10 % [[33], [34], [35]] i.e., if the inclusion of the potential confounder changes the estimate of RERI by 10 % or more, the variable is considered a confounder and should be adjusted for. The analyses revealed that the RERI increased from 2.20 to 2.29 upon the inclusion of the province variable in the confounder set, indicating a change of less than 10 %.

### Statistical analysis

The relative excess risk due to interaction (RERI_RR_) is the primary measure for assessing additive interaction on the risk ratio scale. However, given the rare disease assumption, we used odds ratios instead of risk ratios in this case-control study, i.e., RERI_OR_ = OR_11_ - OR_10_ - OR_01_ + 1 where OR_11_, OR_10_, and OR_01_ are the causal odds ratios comparing subjects with both exposures, with only opium use, and with only cigarette smoking compared with those without any exposure, respectively. If RERI_OR_ is greater than zero, the interaction is often referred to as positive or supper- additive.

The InteractionR package of R software version 4.3.1 (2023-06-16 ucrt) was used for the full reporting of interaction analyses. To estimate OR_11_, OR_10_, and OR_01_, a logistic regression model was fitted using the glm function in R, with bladder cancer as the response variable and opium use, cigarette smoking, and confounders as explanatory variables.

The linearity assumption for the confounding by age was checked via fractional polynomials, LOWESS, and restricted cubic splines using Stata, version 16 (Stata Corp., College Station, TX, licensed to Tampere University) [36,[37], [75]]. The plots of fractional polynomials and LOWESS showed a linear association between age and response variables. The *P*-value for deviance difference comparing the best fractional polynomial with two terms (df = 4) and linear model was 0.364. Also, using the restricted cubic spline with 5 knots located at 0.05, 0.275, 0.5, 0.725, and 0.95 percentiles [38] (37, 52, 59, 65, and 77 years old), the P-value obtained from the likelihood ratio test for the linearity assumption was 0.330.

The concept of sufficient-cause or mechanistic interaction is applicable when there exist individuals, for whom the outcome is only in the presence of both exposures. At the same time, it does not occur with either exposure alone. To examine different mechanistic forms of interaction, including sufficient-cause synergism (the existence of a mechanism whereby certain individuals would encounter the outcome when exposed to both conditions yet would not experience the outcome when subjected to either condition in distinct) and compositional epistasis (the existence of a mechanism whereby certain individuals would the outcome if and only if both exposures were present), RERI_OR_ was used.

However, the presence of super-additive interaction only indicates mechanistic interaction under certain additional assumptions: none of the exposures can be preventive for any individual in the population, often referred to as a positive “monotonicity” assumption.

If RERI_OR_ is greater than 1, it signifies the presence of sufficient-cause synergism. Moreover, a RERI_OR_ value higher than 2 indicates the presence of two types of mechanistic interactions: sufficient-cause synergism and compositional epistasis, without requiring a monotonicity assumption. When it is assumed that two exposures have a positive monotonic effect, a RERI_OR_ above 0 would indicate two forms of mechanistic interactions. On the other hand, when only one exposure is considered, a RERI_OR_ greater than one also suggests the existence of two forms of mechanistic interactions. For the presence of super-additive interaction, a RERI_OR_ greater than zero, sufficient-cause synergism with a RERI_OR_ greater than one, and epistatic interaction with a RERI_OR_ greater than two were assessed. One-sided *P*-values were computed [39,40].

The inverse probability-of-treatment weighting (IPTW) approach was used to estimate the RERI_OR_ and handle the misspecification problem of the logistic regression [[41], [42], [43], [44], [45], [69], [74]]. We fitted two logistic regression models among the controls: 1) a logistic model, regressing opium use on the confounders, and 2) another model, regressing the cigarette smoking on the confounders and opium use. The weights based on each model were the inverse of the received probability of the exposure, W_1_ and W_2_, calculated for cases and controls, and the overall weight was W = W_1_ × W_2_. We used the marginal structural linear odds model:

$$\text{Odds}=\exp\left(\beta_{0}\right)\left(1+\beta_{1}O+\beta_{2}S+\beta_{3}\mathrm{OS}\right)$$

where O and S denote opium use and cigarette smoking. The parameter β_3_ is RERI_OR_, estimated using a weighted least square associational linear odds model, corresponding to the marginal structural model mentioned above [19,41]. We used weight truncation at the optimal level (99.5th percentile) to improve the robustness of the estimation process by reducing the impact of outliers in the data [46]. Valid but conservative 95 % CIs were calculated using robust standard errors [47]. These regression models were fitted using Stata Version 18. All reported *P*-values are derived from one-sided tests using the same methodology mentioned above in the unweighted model, with the significance level set at 0.025 [39,[40], [67], [68]].

The attributing effects of the interaction between opium use and cigarette smoking were assessed [13,[48], [49], [50]]. The R function *additive_interactions* was applied to estimate additive measures and test different hypothesises with a logistic regression model, the glm function in R [51,52].

### Ethical approval

The study was approved by the Ethics Committee of Tehran University of Medical Sciences (Code: IR.TUMS.SPH.REC.1401.270). The IROICAN study was approved by the ethics committee of the National Institute of Medical Research Development (Code: IR.NIMAD.REC.1394.027). All subjects provided their written consent for participation, and the data was kept confidential.

## Results

Of 4194 subjects, 3024 (%72) of them are male. The mean ± SD of age was 58.42 ± 11.74. Thirty-seven percent of cases and controls were included in the study from the cities of participating provinces (Table 1).

**Table 1:** Demographic and clinical features of bladder cancer cases and controls recruited in IROPICAN study in Iran.

Characteristic	Bladder cancer cases	Controls
	*N* = 717	*N* = 3477
	n (%)	n (%)
Age (years); mean (SD)	63.61 (11.1)	57.35 (11.6)
BMI (kg/m2); mean (SD)	25.66 (4.6)	26.74 (6.1)
Residency		
Capital cities	267 (37.2)	1310 (37.7)
Other	450 (62.8)	2167 (62.3)
Male Sex	624 (87.0)	2400 (69.0)
Married	633 (88.3)	3147 (90.5)
SES		
Low	288 (40.2)	974 (28.0)
Moderate	227 (31.7)	1174 (33.8)
High	202 (28.2)	1329 (38.2)
Family History^1^ (yes)	139 (19.4)	713 (20.6)
Cigarette Smoking (yes)	430 (60.0)	977 (28.1)
Opium use (yes)	330 (46.0)	596 (17.0)

1 Family history for all cancers.

We presented the causal interaction of opium use and cigarette smoking on bladder cancer, as outlined in Table 2, based on updated guidelines [22,53]. Our findings indicated that bladder cancer risk was much higher when both opium use and cigarette smoking were used together (adjusted OR = 6.34, 95 % CI 5.02–7.99; *p* < 0.001), and we had evidence for a causal super-additive or positive additive interaction between them (adjusted RERI_OR_ = 2.20, 95 %CI 0.78–3.62; *p* = 0.001).

**Table 2:** Interaction of opium use and cigarette smoking on odds of bladder cancer: a multicenter case-control study in Iran.

	Cigarette smoking		
	No	Yes	ORs (95 %CI) for smoking within strata of opium use
	OR (95 %CI)	OR (95 %CI)	
Opium user			
No	1.0	2.40 (1.88–3.06); *p* < 0.001	2.40 (1.88–3.06); *p* < 0.001
Yes	2.74 (1.95–3.84); p < 0.001	6.34 (5.02–7.99); p < 0.001	2.32 (1.64–3.27); p < 0.001
ORs (95 %CI) for Opium use within strata of smoking	2.74 (1.95–3.84); p < 0.001	2.64 (2.07–3.37); p < 0.001	

The measure of interaction on additive scale: RERI_OR_ (95 %CI) = 2.20 (0.78–3.62); *P*-values: supper-additive = 0.001, sufficient-cause synergism = 0.048, epistatic interaction = 0.390.

The measure of interaction on the additive scale: Weighted RERI_OR_ (from Marginal Structural Linear Odds Model) (95 %CI) = 2.02 (0.47–3.58); *P*-values: supper-additive = 0.005, sufficient-cause synergism = 0.099, epistatic interaction = 0.489.

Measure of interaction on Multiplicative scale: Ratio of ORs (95 %CI) = 0.96 (0.63–1.46); *p* = 0.867.

Note: ORs are adjusted for age, sex, socio-economic status, and passive smoking; all reported *P*-values are derived from one-sided tests, with the significance level set at 0.025, without imposing monotonicity assumption.

The marginal structural linear odds model analysis revealed a significant super-additive interaction (weighted RERI_OR_ = 2.02, 95 %CI 0.47–3.58; *p* = 0.005). We also checked the multiplicative interaction between opium use and cigarette smoking on odds of bladder cancer, which did not show sufficient evidence to support the presence of an interaction on a multiplicative scale (adjusted ORs = 0.96, 95 % CI 0.63–1.46; *p* = 0.867).

The multiple logistic regression model assessed different mechanistic interaction forms of opium use and cigarette smoking. According to the result of the model, we did not find strong evidence for a sufficient-cause synergism (*P*-value = 0.048) and no compositional epistasis interaction (*P*-value = 0.390) between opium use and cigarette smoking without imposing the monotonicity assumption. When we assumed monotonicity for two exposures, we found a sufficient-cause synergism (*P*-value = 0.001) and compositional epistasis interaction (P-value = 0.001) between exposures. The IPTW approach also did not show evidence of a mechanistic interaction, sufficient-cause synergism (P-value = 0.099), or compositional epistasis interaction (P-value = 0.489). However, when two exposures are considered to have a monotonic effect, the findings indicate the presence of mechanistic interactions (P-value = 0.005).

In Table 3, attributing effects to the interaction of opium use and cigarette smoking on bladder cancer are reported. According to the findings, the synergy index (SI) also showed a positive additive interaction between two exposures (SI (95 %CI) = 1.70 (1.18–2.44); *p* = 0.004) that reflects the extent to which the ORs for both exposures together exceed one. In this case, the combined effect is 1.7 times what would be expected if the effects of opium use and cigarette smoking were added.

**Table 3:** Attributing effects to interactions between opium use and cigarette smoking on bladder cancer a multicenter case-control study in Iran.

Index	Value^1^	95 % CI	*P*-value
Synergy; (OR_11_–1) / ((OR_10_–1) + (OR_01_–1))	1.70	1.18 to 2.44	0.004
Attributable Proportion^2^; (RERI_OR_) / (OR_11_)	0.34	0.16 to 0.53	<0.001
Proportion of joint effect due to interaction; (RERI_OR_) / (OR_11_–1)	0.41	0.20 to 0.62	<0.001
Proportion of joint effects due to Opium use; (OR_10_–1) / (OR_11_–1)	0.32	0.15 to 0.49	<0.001
Proportion of joint effects due to cigarette smoking; (OR_01_–1) / (OR_11_–1)	0.26	0.16 to 0.36	<0.001

Note: OR_11_, OR_10_, and OR_01_ are the causal odds ratios comparing subjects with both exposures, with only opium use, and with only cigarette smoking compared with those without any exposure, respectively; RERI_OR_ is a relative excess risk due to interaction.

1 Adjusted for age, sex, socio-economic status, passive smoking.

2 Proportion of bladder cancer risk in the doubly-exposed group attributable to the interaction.

We calculated other measures of additive interaction, and analyses revealed 34 % (95 %CI = 16 % - 53 %) of the bladder cancer risk in the doubly exposed group due to the interaction itself. Another version of the attributable proportion showed 41 % (95 %CI = 20 % - 62 %) of joint effects attributable to the interaction of opium use and cigarette smoking.

## Discussion

We identified bladder cancer risk significantly increased when opium uses and cigarette smoking happened together, showing a super-additive and mechanistic interaction between these exposures when two exposures have monotonic effects.

The presence of a super-additive interaction between these exposures suggests that an intervention targeting cigarette smokers who use opium would yield greater benefits compared to those who do not [13,17]. Also, other surrogate measures of additive interactions, such as the synergy index, confirmed our result. Some studies suggested that the synergy index is a better option to assess the additive interaction of exposures because of the low uniqueness problem [16], but Vanderweele and his colleagues believe that weighted RERI handles these problems. It is also a desirable measure for assessing mechanistic interactions [41].

Moreover, in the presence of a mechanistic interaction, there must be a sufficient-component cause for bladder cancer, which has both opium use and cigarette smoking as components according to the Rothman's sufficient-component cause framework [54]. Even though the positive monotonicity assumption is strong, it is highly unlikely that one would consider smoking or opium use to be protective against bladder cancer for any individual. While it is possible that some individuals may develop bladder cancer as a result of smoking or opium use, and others may experience no effect, the notion that smoking or opium use could prevent bladder cancer in any individual is not tenable. In other words, it is improbable to assert that an individual would avoid bladder cancer due to smoking or opium use, while they would develop the disease in the absence of smoking and opium use. Consequently, the assumption of positive monotonicity regarding the impact of smoking and opium use on bladder cancer appears to be a plausible hypothesis. While numerous studies have highlighted the effect of opium use [9,55] and cigarette smoking [[56], [57], [58]] on bladder cancer are consistent with our findings, we found no study reporting a causal additive interaction of these exposures on bladder cancer development.

Previous studies showed that dual users of opium use and tobacco smoking were exposed to many toxicants and carcinogens [59]. The mechanism of carcinogenicity primarily involves the direct and indirect interactions of chemical compounds found in these substances with cellular DNA. In the case of cigarettes, the smoke contains a complex mixture of over 7000 chemicals, including at least 70 known carcinogens such as benzene, formaldehyde, and polycyclic aromatic hydrocarbons (PAHs). These chemicals can induce DNA damage directly by forming DNA adducts or indirectly by producing reactive oxygen species that oxidize DNA, proteins, and lipids, thereby disrupting cellular function and promoting malignant transformation [60]. Similarly, opium, which contains a range of alkaloids such as morphine and codeine, has been linked to cancer through chronic inflammation pathways and immunosuppressive effects [61]. A combination of these factors contributes significantly to the increased risk of cancers, particularly lung, bladder, and oesophageal cancers in smokers and gastrointestinal cancers in opium users [7,60].

Hadji and her colleagues recently examined the joint effect of opium use and tobacco smoking (cigarette, water pipe, pipe, chewing tobacco, Chopogh) and found a significant RERI. However, they had some limitations in the analysis, including inappropriate adjustment of confounders in the model, a lack of testing for mechanistic interactions, and the use of the conventional statistic method [9].

Our analyses assessed two forms of mechanistic interactions, sufficient-cause synergism and compositional epistasis, between opium use and cigarette smoking. Although we demonstrated a statistical super-additive interaction and mechanistic interactions between these exposures, our findings emphasize that neither statistical additive interaction nor sufficient-cause interaction nor epistatic interaction necessarily offers insight into physical or functional interactions [13]. One of the key strengths of this paper is controlling confounder factors to estimate causal additive interaction measures using cDAGs [26,62]. The large sample size, comprising cases from the ten provinces of Iran, has increased the generalizability of our results and reduced the random error, resulting in narrow confidence intervals for interaction parameters. Also, using a large number of the controls collected in the IROPICAN study increased the statistical power of the study. Furthermore, analyses were conducted to calculate all proposed measures of additive interaction in the literature, such as RERI, APs, SI, the Proportion of the joint effect due to each exposure, and mechanistic forms of interaction [13].

While our study sheds light on the effects of opium uses and cigarette smoking on bladder cancer, it is important to acknowledge the limitations of our current research. Case-control studies can be affected by systematic errors due to inaccurate measurement of the exposures as well as bias in selecting the controls [[70], [71], [72]]. Another limitation in all observational studies, including current study is unmeasured confounding though we have adjusted minimally sufficient set of confounders using causal diagrams [73]. Lifestyle risk factors such as physical activity are associated with cigarette smoking and opium use with possible feedbacks between them, i.e. physical activity may be affected by cigarette smoking and opium use, and also may affect these exposures. Given the concurrent measurements of these variables and considering that the mediation role of the physical activity is much stronger than its possible confounding role, the arrows between the variable and exposures have not been shown in Fig. 1, and to avoid over-adjustment bias, it has not been adjusted for in the analysis. We have attempted to diagnose outcomes by pathological results and measure exposures accurately using validated questionnaires [20]. Nevertheless, one avenue for future exploration could involve investigating interactions in large cohort studies. Also, advanced statistical approaches such as doubly robust and Bayesian would be helpful [63].

In conclusion, there is a causal additive interaction between opium use and cigarette smoking. We observed a super-additive interaction, suggesting the need to focus interventions on specific subgroups. Furthermore, the presence of mechanistic interactions offers profound insights into the mechanisms of cancer induction.

## CRediT authorship contribution statement

**Rahim Akrami:** Conceptualization, Methodology, Project administration, Software, Validation, Formal analysis, Investigation, Writing – original draft, Visualization. **Maryam Hadji:** Conceptualization, Methodology, Project administration, Investigation, Data curation, Writing – review & editing. **Hamideh Rashidian:** Investigation, Methodology, Project administration, Data curation, Writing – review & editing. **Maryam Nazemipour:** Formal analysis, Software, Validation, Writing – review & editing. **Ahmad Naghibzadeh-Tahami:** Investigation, Project administration, Writing – review & editing. **Alireza Ansari-Moghaddam:** Investigation, Project administration, Writing – review & editing. **Kazem Zendehdel:** Conceptualization, Supervision, Methodology, Project administration, Resources, Funding acquisition, Writing – review & editing. **Mohammad Ali Mansournia:** Conceptualization, Supervision, Project administration, Validation, Methodology, Software, Formal analysis, Writing – review & editing.

## Declaration of competing interest

The authors declare that they have no known competing financial interests or personal relationships that could have appeared to influence the work reported in this paper.

## References

[1] Global Cancer Observatory (2024). https://gco.iarc.fr/today.

[2] Shakhssalim N., Hosseini S.Y., Basiri A. (2010). Prominent bladder cancer risk factors in Iran. Asian Pac J Cancer Prev.

[3] Westhoff E., Maria de Oliveira-Neumayer J., Aben K.K. (2016). Low awareness of risk factors among bladder cancer survivors: new evidence and a literature overview. Eur J Cancer.

[4] Cumberbatch M.G.K., Jubber I., Black P.C. (2018). Epidemiology of bladder cancer: a systematic review and contemporary update of risk factors in 2018. Eur Urol.

[5] Freedman N.D., Abnet C.C., Caporaso N.E. (2016). Impact of changing US cigarette smoking patterns on incident cancer: risks of 20 smoking-related cancers among the women and men of the NIH-AARP cohort. Int J Epidemiol.

[6] Rahimi-Movaghar A., Gholami J., Amato L. (2018). Pharmacological therapies for management of opium withdrawal. Cochrane Database Syst Rev.

[7] Bidary M.Z., Sahranavard M., Rezayat A.A. (2021). Opium as a carcinogen: a systematic review and meta-analysis. EClinicalMedicine.

[8] Carcinogenicity of opium consumption (2020). Lancet Oncol.

[9] Hadji M., Rashidian H., Marzban M. (2022). Opium use and risk of bladder cancer: a multi-centre case-referent study in Iran. Int J Epidemiol.

[10] Sheikh M., Shakeri R., Poustchi H. (2020). Opium use and subsequent incidence of cancer: results from the Golestan cohort study. Lancet Glob Health.

[11] Mansouri M., Naghshi S., Parsaeian M. (2022). Opium use and cancer risk: a comprehensive systematic review and meta-analysis of observational studies. Int J Clin Pract.

[12] Simonds N.I., Ghazarian A.A., Pimentel C.B. (2016). Review of the gene-environment interaction literature in cancer: what do we know?. Genet Epidemiol.

[13] VanderWeele T.J., Knol M.J. (2014). A tutorial on interaction. Epidemiol Methods.

[14] Kamangar F. (2012). Effect modification in epidemiology and medicine. Arch Iran Med.

[15] Rothman K.J., Greenland S., Walker A.M. (1980). Concepts of interaction. Am J Epidemiol.

[16] Skrondal A. (2003). Interaction as departure from additivity in case-control studies: a cautionary note. Am J Epidemiol.

[17] Greenland S. (2009). Interactions in epidemiology: relevance, identification, and estimation. Epidemiology.

[18] VanderWeele T.J., Vansteelandt S. (2014). Invited commentary: some advantages of the relative excess risk due to interaction (RERI)–towards better estimators of additive interaction. Am J Epidemiol.

[19] Vanderweele T.J., Vansteelandt S., Robins J.M. (2010). Marginal structural models for sufficient cause interactions. Am J Epidemiol.

[20] Hadji M., Rashidian H., Marzban M. (2021). The Iranian study of opium and cancer (IROPICAN): rationale, design, and initial findings. Arch Iran Med.

[21] Rashidian H., Hadji M., Marzban M. (2017). Sensitivity of self-reported opioid use in case-control studies: healthy individuals versus hospitalized patients. PloS One.

[22] Knol M.J., VanderWeele T.J. (2012). Recommendations for presenting analyses of effect modification and interaction. Int J Epidemiol.

[23] VanderWeele T.J. (2009). On the distinction between interaction and effect modification. Epidemiology.

[24] Textor J., van der Zander B., Gilthorpe M.S. (2017). Robust causal inference using directed acyclic graphs: the R package ‘dagitty’. Int J Epidemiol.

[25] Mansournia M.A., Hernán M.A., Greenland S. (2013). Matched designs and causal diagrams. Int J Epidemiol.

[26] Etminan M., Collins G.S., Mansournia M.A. (2020). Using causal diagrams to improve the design and interpretation of medical research. Chest.

[27] Mansournia M.A., Higgins J.P.T., Sterne J.A.C. (2017). Biases in randomized trials: a conversation between trialists and epidemiologists. Epidemiology.

[28] Kyriacou D.N., Greenland P., Mansournia M.A. (2023). Using causal diagrams for biomedical research. Ann Emerg Med.

[29] Etminan M., Brophy J.M., Collins G. (2021). To adjust or not to adjust: the role of different covariates in cardiovascular observational studies. Am Heart J.

[30] Mansournia M.A., Nazemipour M., Etminan M. (2022). Interaction contrasts and collider bias. Am J Epidemiol.

[31] Mansournia M.A., Nazemipour M., Etminan M. (2021). Causal diagrams for immortal time bias. Int J Epidemiol.

[32] Etminan M., Nazemipour M., Candidate M.S. (2021). Potential biases in studies of acid-suppressing drugs and COVID-19 infection. Gastroenterology.

[33] Mansournia M.A., Jewell N.P., Greenland S. (2018). Case–control matching: effects, misconceptions, and recommendations. Eur J Epidemiol.

[34] Mansournia M.A., Poole C. (2023). Case–control matching on confounders revisited. Eur J Epidemiol.

[35] Mickey R.M., Greenland S. (1989). The impact of confounder selection criteria on effect estimation. Am J Epidemiol.

[36] Mansournia M.A., Collins G.S., Nielsen R.O. (2021). A CHecklist for statistical assessment of medical papers (the CHAMP statement): explanation and elaboration. BJSM.

[37] Mansournia M.A., Nazemipour M. (2024). Recommendations for accurate reporting in medical research statistics. Lancet.

[38] Frank E.H. (2015).

[39] Greenland S., Mansournia M.A., Joffe M. (2022). To curb research misreporting, replace significance and confidence by compatibility: a preventive medicine Golden Jubilee article. Prev Med.

[40] Mansournia M.A., Nazemipour M., Etminan M. (2022). P-value, compatibility, and S-value. Glob Epidemiol.

[41] VanderWeele T.J., Vansteelandt S. (2011). A weighting approach to causal effects and additive interaction in case-control studies: marginal structural linear odds models. Am J Epidemiol.

[42] Mansournia M.A., Etminan M., Danaei G. (2017). Handling time varying confounding in observational research. BMJ.

[43] Mansournia M.A., Naimi A.I., Greenland S. (2018). The implications of using lagged and baseline exposure terms in longitudinal causal and regression models. Am J Epidemiol.

[44] Mansournia M.A., Altman D.G. (2016). Inverse probability weighting. BMJ.

[45] Mansournia M.A., Danaei G., Forouzanfar M.H. (2012). Effect of physical activity on functional performance and knee pain in patients with osteoarthritis: analysis with marginal structural models. Epidemiology.

[46] Xiao Y., Moodie E.E.M., Abrahamowicz M. (2013). Comparison of approaches to weight truncation for marginal structural cox models. Epidemiol Methods.

[47] Mansournia M.A., Nazemipour M., Naimi A.I. (2020). Reflection on modern methods: demystifying robust standard errors for epidemiologists. Int J Epidemiol.

[48] VanderWeele T.J., Tchetgen Tchetgen E.J. (2014). Attributing effects to interactions. Epidemiology.

[49] VanderWeele T.J. (2013). Reconsidering the denominator of the attributable proportion for interaction. Eur J Epidemiol.

[50] Abdollahpour I., Salimi Y., Nedjat S. (2023). Additive interaction between dietary inflammatory index and some key risk factors of multiple sclerosis: a population-based incident case–control study. Nutr Neurosci.

[51] Mathur M.B., VanderWeele T.J. (2018). R function for additive interaction measures. Epidemiology.

[52] (2024). R: a language and environment for statistical computing [program]. 4.3.1 version.

[53] Vandenbroucke J.P., von Elm E., Altman D.G. (2014). Strengthening the reporting of observational studies in epidemiology (STROBE): explanation and elaboration. Int J Surg.

[54] Rothman K.J. (1995). Causes. 1976. Am J Epidemiol.

[55] Afshari M., Janbabaei G., Bahrami M.A. (2017). Opium and bladder cancer: a systematic review and meta-analysis of the odds ratios for opium use and the risk of bladder cancer. PloS One.

[56] van Osch F.H., Jochems S.H., van Schooten F.J. (2016). Quantified relations between exposure to tobacco smoking and bladder cancer risk: a meta-analysis of 89 observational studies. Int J Epidemiol.

[57] Masaoka H., Matsuo K., Ito H. (2016). Cigarette smoking and bladder cancer risk: an evaluation based on a systematic review of epidemiologic evidence in the Japanese population. Jpn J Clin Oncol.

[58] Cumberbatch M.G., Rota M., Catto J.W.F. (2016). The role of tobacco smoke in bladder and kidney carcinogenesis: a comparison of exposures and meta-analysis of incidence and mortality risks. Eur Urol.

[59] Etemadi A., Poustchi H., Calafat A.M. (2020). Opiate and tobacco use and exposure to carcinogens and toxicants in the Golestan cohort study. Cancer Epidemiol Biomarkers Prev.

[60] Hecht S.S. (2003). Tobacco carcinogens, their biomarkers and tobacco-induced cancer. Nat Rev Cancer.

[61] Vallejo R., de Leon-Casasola O., Benyamin R. (2004). Opioid therapy and immunosuppression: a review. Am J Ther.

[62] Greenland S., Pearl J., Robins J.M. (1999). Causal diagrams for epidemiologic research. Epidemiology.

[63] Chu H., Nie L., Cole S.R. (2011). Estimating the relative excess risk due to interaction: a bayesian approach. Epidemiology.

[64] Mansournia M.A., Nazemipour M., Etminan M. (2022). Time-fixed vs time-varying causal diagrams for immortal time bias. Int. J. Epidem..

[65] Mansournia M.A., Higgins J.P.T., Sterne J.A.C., Hernán M.A. (2017). Biases in randomized trials: a conversation between trialists and epidemiologists. Epidemiology.

[66] Taheri Soodejani M., Tabatabaei S.M., Lotfi M.H., Nazemipour M., Mansournia M.A. (2023). Adjustment for collider bias in the hospitalized Covid-19 setting. Glob. Epidem..

[67] Rovetta A., Mansournia M.A., Vitale A. (2024). For a proper use of frequentist inferential statistics in public health. Glob. Epidem..

[68] Rovetta A., Mansournia M.A. (2024). P>0.05 Is Good: The NORD-h Protocol for Several Hypothesis Analysis Based on Known Risks, Costs, and Benefits. Journal of preventive medicine and public health = Yebang Uihakhoe chi.

[69] Smith M.J., Mansournia M.A., Maringe C. (2022). Introduction to computational causal inference using reproducible Stata, R, and Python code: A tutorial. Statistics in Medicine.

[70] Pakzad R, Nedjat S, Salehiniya H (2023). Effect of alcohol consumption on breast cancer: probabilistic bias analysis for adjustment of exposure misclassification bias and confounders. BMC Medic. Res. Methodol..

[71] Pakzad R., Nedjat S., Yaseri M. (2020). Effect of Smoking on Breast Cancer by Adjusting for Smoking Misclassification Bias and Confounders Using a Probabilistic Bias Analysis Method. Clinic. Epidem..

[72] Malekifar P., Nedjat S., Abdollahpour I., Nazemipour M., Malekifar S., Mansournia M.A. (2023). Impact of Alcohol Consumption on Multiple Sclerosis Using Model-based Standardization and Misclassification Adjustment Via Probabilistic Bias Analysis. Archives of Iranian medicine.

[73] Navadeh S., Mirzazadeh A., McFarland W. (2020). Unsafe Injection Is Associated with Higher HIV Testing after Bayesian Adjustment for Unmeasured Confounding. Archives of Iranian medicine.

[74] Koohi F., Khalili D., Soori H., Nazemipour M., Mansournia M.A. (2022). Longitudinal effects of lipid indices on incident cardiovascular diseases adjusting for time-varying confounding using marginal structural models: 25 years follow-up of two US cohort studies. Glob. Epidem..

[75] Mansournia M.A., Collins G.S., Nielsen R.O. (2021). CHecklist for statistical Assessment of Medical Papers: the CHAMP statement. British journal of sports medicine.

